# Differential impedance spectra analysis reveals optimal actuation frequency in bulk mode acoustophoresis

**DOI:** 10.1038/s41598-019-55333-1

**Published:** 2019-12-13

**Authors:** Valentina Vitali, Giulia Core, Fabio Garofalo, Thomas Laurell, Andreas Lenshof

**Affiliations:** Department of Biomedical Engineering, Lund University, Lund, Sweden

**Keywords:** Physics, Electrical and electronic engineering, Biomedical engineering

## Abstract

This work reports a method to select the optimal working frequency in transversal bulk resonator acoustophoretic devices by electrical impedance measurements. The impedance spectra of acoustophoretic devices are rich in spurious resonance peaks originating from different resonance modes in the system not directly related to the channel resonance, why direct measurement of the piezoelectric transducer impedance spectra is not a viable strategy. This work presents, for the first time, that the resonance modes of microchip integrated acoustophoresis channels can be identified by sequentially measuring the impedance spectra of the acoustophoretic device when the channel is filled with two different fluids and subsequently calculate the Normalized Differential Spectrum (NDS). Seven transversal bulk resonator acoustophoretic devices of different materials and designs were tested with successful results. The developed method enables a rapid, reproducible and precise determination of the optimal working frequency.

## Introduction

Acoustophoresis has been demonstrated to be an efficient, gentle and label-free method for manipulation of suspended particles and cells in microfluidic devices^1–3^. The technology is based on the principle that particulate matter in a sound field are affected by acoustic radiation forces. These forces transport the matter to the acoustic standing wave node or antinode depending on physical properties (density and compressibility) of the particles as compared to the surrounding medium^4–7^. A basic unit operation of acoustophoresis is the precise alignment and focusing of particles in a microfluidic channel^8,9^. Starting from this principle, different applications have emerged including separation^10–15^, concentration^3,16,17^, washing^18^, medium exchange^19^, and trapping^20,21^ of particles and cells.

To efficiently combine acoustics and microfluidics, acoustofluidic devices must be designed as proper acoustic resonators^22^. Several device designs have been suggested in the past decades, such as standing surface acoustic wave devices (SSAW)^23–25^, layered resonators^18,26^ and transversal resonator devices^27,28^. Although the choice of material, the geometrical parameters and strategies of ultrasonic actuation are different, they all rely on the generation of a standing wave or resonance condition in the microchannel in order to spatially localize particles. Transversal resonator devices, in particular, utilize bulk acoustic waves generated by a piezoelectric transducer (piezo) to create a standing wave in the direction orthogonal to the actuation, that is commonly the width of the microfluidic channel. The frequency of the signal applied to the piezo must be tuned to generate an integer number of half wavelength across the width of the microchannel, such that a standing wave with well-defined pressure nodes and pressure maxima forms^29^. Under these conditions, a resonance builds up in the channel and particles can be precisely moved and spatially manipulated. It is therefore necessary for transversal resonators to precisely identify the frequencies at which the microchannel is in resonance, i.e. the best working frequencies.

When designing a transversal resonator, the microchannel resonance frequency is typically determined using a one-dimensional standing planar acoustic wave approximation, knowing the channel width (w) and the speed of sound in the sample fluid (c) (Eq. 1),

1 $${\rm{f}}=\frac{{\rm{c}}}{2{\rm{w}}}\,{\rm{n}}$$

where n stands for an integer multiple of the acoustic wavelength. However, in practice, even if the microchannel width is designed to support an integer multiple of the wavelength of the ultrasound, the real microchannel resonance frequency is usually achieved at a slightly different frequency. This is due to the coupling to chip bulk material resonances, variable chip geometries and the actuator mounting. A time-consuming calibration procedure and an experimental characterization of each device is hence necessary to determine the optimal working frequency^30^.

Different techniques have been suggested to identify the best working conditions for acoustophoretic devices, including particle image velocimetry (PIV)^31^, precise temperature and media control^32^, microscopy and laser interferometry^33^. However, these techniques need a skilled operator and do not allow an automatic tuning of the best working frequency during operation.

To overcome these obstacles, selection of the frequency based on electrical measurements has been suggested^34–36^. One of the first steps in this direction was taken by Hawkes *et al*.^37^, who characterized water-filled layered resonator devices by electrical admittance measurements. They concluded that there is a relation between the optimal working frequency and admittance measurements. However, Dual *et al*. pointed out that, given the complexity of these systems, the identification of the microchannel resonance mode by means of electrical measurements is not obvious^30^. Following this, Hammarström *et al*. developed an automatic system to determine the optimal trapping frequency by electrical impedance measurements in simple glass capillary devices used in acoustic trapping applications^38^. Later on, Garofalo *et al*.^29^ proposed an empirical rule to identify the optimal working frequency in more complex transversal resonator devices by electrical measurements analysis, based on theoretical simulations. More recently, Kalb *et al*.^32^ concluded that impedance measurements alone cannot characterize the optimal working frequency in transversal acoustophoretic devices. In particular, they showed that although there is a conductance maximum related to the optimal working frequency, the spectra present additional conductance maxima related to other system resonance modes that cannot be distinguished from the optimal frequency.

To address these shortcomings, we propose a new method that allows for the identification of the optimal working frequency in bulk transversal resonator acoustophoretic devices by an alternative electrical impedance measurement approach. Although the impedance spectrum is rich in large spurious resonance peaks, originating from bulk resonances not related to the channel resonance, we were able to distinguish the peak related to the optimal working frequency through differential impedance analysis. Two fluids, distilled water and 20% Cesium Chloride solution, were infused in the microchannel and impedance measurements were sequentially recorded in the frequency range 1.5–2.8 MHz. The presence of different fluids in the microchannel induced a change in the optimal working condition that could be detected by electrical impedance measurements. The shift in optimal working condition is presented as the absolute value of the complex impedance difference, normalized to the absolute value of the water filled channel impedance spectrum. This enables identification of which impedance peak is related to the microchannel resonance frequency. Seven transversal resonator acoustophoretic devices of different materials and designs were tested with successful results.

The presented method, enables a rapid, reproducible and precise determination of the optimal working frequency, opening the route for an automatic tuning of acoustophoretic devices.

## Results and Discussion

### Impedance measurements variations due to the load

**Figure 1:**
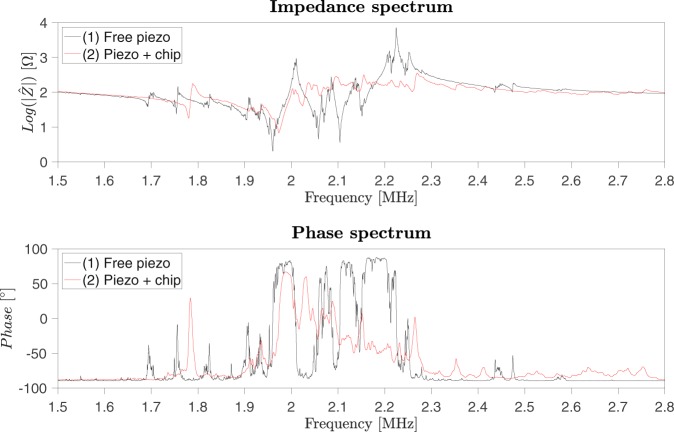
Electrical impedance magnitude and phase spectrum of a piezoelectric transducer at different mechanical loads. Two cases are investigated: (1) free piezoelectric transducer and (2) piezoelectric transducer + water-filled chip (chip S2).

In order to study the impedance magnitude and phase spectra of acoustophoresis manifolds, it was firstly investigated how the spectra change while assembling the devices. Two cases are exemplified here: the free piezoelectric transducer and the piezoelectric transducer attached to the chip. Figure 1 shows the impedance measurements of (1) a free piezoelectric transducer (PZT 26, 1 × 5 × 25 mm) and (2) a piezoelectric transducer attached to a water-filled chip (chip S2). The measurements were carried out in the range from 1.5 MHz to 2.8 MHz.

The impedance measurements of the free piezoelectric transducer clearly show the intrinsic resonance modes of the transducer of which the resonance peak around 1.96 MHz represent the fundamental half wavelength thickness resonance mode. At this frequency, the vibration amplitude is the largest and the conversion of electrical energy into mechanical energy becomes more efficient. The other resonance peaks, characterized by smaller amplitudes, are related to fundamental resonance modes in other directions and/or harmonics thereof.

When the piezoelectric transducer is attached to the chip, the impedance magnitude and phase spectrum are greatly affected. More specifically, the transducer resonance peaks become significantly attenuated and several new resonance peaks related to resonance modes in the chip/transducer assembly emerge. The majority of these modes are not linked to the channel resonance. Using impedance measurements alone, under these conditions, it is not possible to identify which of the peaks that are specifically related to the microchannel resonance frequency^30,32^. However, by doing differential impedance measurements where only the resonance condition of the channel is modified, spurious bulk resonance modes from the chip/transducer assembly can be discriminated.

### Impact of channel medium on the impedance spectrum

Fluids with different speed of sound were utilized in order to modify the acousto-mechanical characteristics of the microchannel. Three fluids were initially chosen: channel filled with (1) air, (2) water and (3) a 20% CsCl solution. In particular, the CsCl solution was chosen to have a concentration of 20%, inducing a change of 0.8% in speed of sound as compared to water.

**Figure 2:**
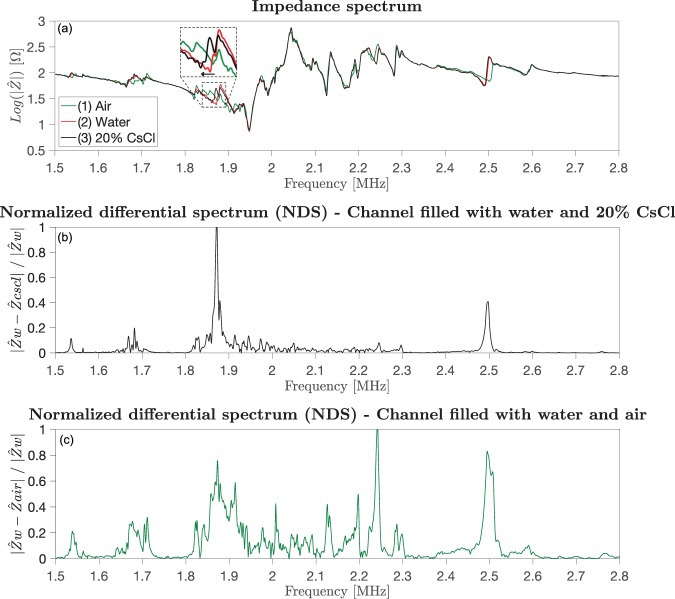
(**a**) Impedance magnitude spectrum of an acoustophoretic device (chip G1) with different media inside the microchannel: (1) air, (2) water and (3) 20% CsCl solution. The measurements show differences in local regions of the spectra, indicating that the channel medium affects the system electrical response. A zoom - in highligths the region of the spectra where there is a significant deviation between the spectra. The deviation can be seen as a frequency shift in the direction indicated by the arrow and is related to the changed resonance frequency linked to the corresponding media loaded in the microchannel. (**b**) Normalized Differential Spectrum (NDS) water vs 20% CsCl. (**c**) NDS water vs air. It can be seen that the NDS water vs 20% CsCl presents two clear peaks. Instead, the noisier NDS water vs air does not give any clear indication of a channel resonance.

Figure 2 shows the impedance magnitude of the device G1 for three conditions: air filled (in green), water filled (in red) and 20% CsCl filled (in black) microchannel. It can be noticed that the measurements overlap in most regions of the spectrum, while significant deviations can be observed in the zoomed-in regions of the plot. More specifically, the impedance spectra of the 20% CsCl filled channel (black line – condition (3)) shows exactly the same profile as the spectrum for the system with a water filled channel (red line – condition (2)), except in a region around 1.87 MHz, where they clearly deviate (main deviation). The impedance spectra of the air filled channel (green line – condition (1)), on the other hand, also presents large deviations compared to the water filled channel, e.g. at 2.25 MHz and 2.5 MHz. The same observations were seen for all the chips in the study. Supplementary Fig. S1a–c show the corresponding impedance spectra of devices S2, S3 and S5 respectively.

Giving extra attention to the regions of the plots where clear deviations of the spectra occur, it can be noticed that the impedance spectra deviate in a similar way. This could be seen as horizontal shifts of the resonance peak, i.e. the resonance peaks of the fluid filled channel shifts from a higher frequency to lower when switching from water to 20% CsCl. An explanation of the horizonal shift of the spectra in conditions (2)-(3) is related to the difference in microchannel resonance frequency in the two cases. As previously reported it is the speed of sound in the fluid that dominates the resonance shift of the microchannel^32^. Neglecting for a moment the air-filled channel and considering only the water and 20% CsCl filled channel conditions, 2 and 3, the speed of sound in water (1498 m/s at 25 °C) is higher than the one in the 20% CsCl solution (1486 m/s at 25 °C). Consequently, when the speed of sound decreases, going from water to 20% CsCl, the resonance frequency of the microchannel decreases, (Eq. 1). This shift in the resonance frequency follows the direction indicated by the horizontal arrow in the zoomed-in region in Fig. 2a. In contrast, the electrical response of the air filled device, condition (1) represents only the bulk material and does not give any information about the microchannel.

The impedance and phase spectra acquired with different fluids inside the microchannel show regional deviations and since the only change introduced into the transducer/chip assembly was the fluid infused into the channel, the deviations in the spectra was assumed to be related to the presence of different fluids inside the system. It was thus hypothesized that it could be possible to derive information about the microchannel resonance solely from the electrical response of the entire system.

**Figure 3:**
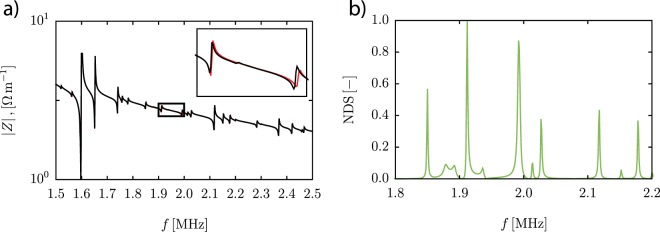
(**a**) Modelled impedance spectra for a typical piezoceramic/chip assembly. Black line CsCl filled channel, Red line water filled channel. (**b**) Normalized Differential Spectrum (NDS) derived from (**a**).

The experimental observations that show significant impedance spectra differences in the region of the theoretical channel resonance frequency, Eq. 1, when altering the fluid (H_2_O vs. CsCl) were also observed when modelling a typical piezoceramic/chip assembly. In line with the experiments (Fig. 2), the modelled impedance spectra as shown in Fig. 3a displayed the largest alterations in the frequency region close to the channel resonance (channel width 375 μm). This is more clearly seen in Fig. 3b, showing the corresponding Normalized Differential Spectrum (NDS) using the 2D-model previously presented by Garofalo *et al*.^29^. The cleaner spectra derived from the modelling as compared to the experimental data is attributed to the modelling being based on a 2D-resonance model, thus not accounting for resonances in full 3D. Since the mass of the fluid in the channel only comprises 0.1% of the total system (calculated from the cross-section dimensions and the material properties of the assembly), it can be anticipated that the impact of the energy in the channel relative the total system energy can only play a role in the impedance readout in frequency ranges close to a channel resonance mode. It can be noted that the piezo electric actuator comprises 78% of the mass in the system as compared to 0.1% for the fluid in the channel. Hence the piezo actuator dominates the system response why the channel resonance should have a negligible impact on the overall system response.

### Identifying the optimal microchannel resonance frequency

#### Computation of the normalized differential spectrum

The relation between the deviations in the spectra and the microchannel resonance frequency as a function of different media in the channel was further investigated. First the complex-valued impedance was calculated as

2 $$\hat{Z}=|\hat{Z}|exp(\frac{i\varphi \pi }{180^\circ })$$

where $|\hat{Z}|$ is the impedance magnitude and φ is the phase angle. Then the Normalized Differential Spectrum (NDS) was calculated as the absolute value of the difference between the complex-valued impedance of the water filled and either the air filled (water vs. air) or 20% CsCl filled channel (water vs. 20% CsCl), normalized against the water filled channel impedance magnitude. An NDS peak is obtained at the frequency of the maximum impedance deviation in regions where impedance spectra differences are observed.

#### Analysis of the NDS of water vs air and water vs 20% CsCl

The NDS of water vs air and water vs. 20% CsCl were compared. Figure 2 and Supplementary Fig. S2 show the impedance magnitude spectrum, the NDS of the channel with water and 20% CsCl filled and with water and air for chip G1 and S5 respectively.

For both chip geometries it was found that the NDS of water vs air and water vs 20% CsCl displayed significantly different profiles. The NDS of water vs. 20% CsCl of chip G1 (Fig. 2b) reveals a distinct NDS peak around 1.87 MHz and a lower differential peak around 2.5 MHz. The corresponding NDS for chip S5 displayed a sharp peak at 2.35 MHz (water vs. 20% CsCl), Fig. S2b. Whereas the NDS of water vs air in both cases, Figs. 2c & S2c displayed a significantly noisier profile. Similar observations were made for all investigated chip designs. Our interpretation of this result is that the spectra deviations of a channel filled with CsCl as compared to water better reflects the resonant properties of the channel rather than the entire system. The noisier profile of the NDS for water vs. air was interpreted as a result of multiple major bulk mode resonance changes, as the air in the channel does not conduct or support bulk mode resonances which in contrast are coupled through the channel when filled with a liquid. Hence, in order to define a method to select the optimal working frequency for acoustophoretic devices, the NDS of water vs air was discarded and only the NDS based on the complex valued impedances of water and 20% CsCl filled channel was considered for the further analysis. As reasoned above, it was hypothesized that the peaks in the NDS, and thus the deviations in the spectra between the water and 20% CsCl curve, were related to the microchannel resonance frequency and consequently that efficient acoustophoretic particle focusing was expected at the NDS peak frequencies.

#### Comparing the NDS to FocuScan data

**Figure 4:**
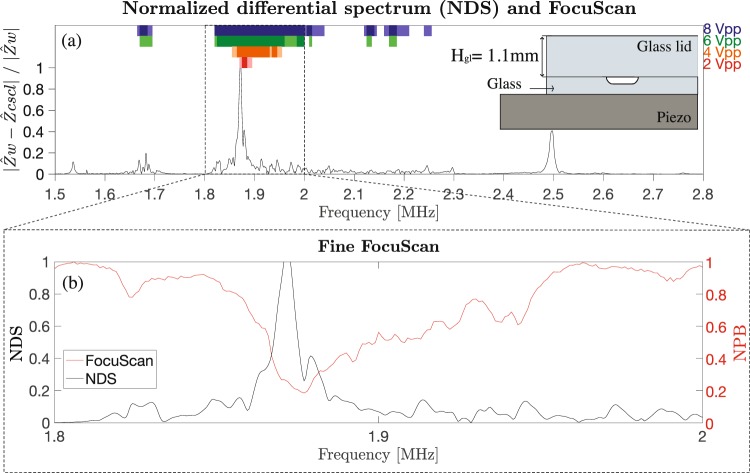
(**a**) Normalized differential spectrum (NDS) water vs 20% CsCl (chip G1). Colored bars at the top indicate the level of focusability measured with the FocuScan method at actuation voltages from 2–8 Vpp. Light color bars indicate moderate acoustic particle focusing, and intense colored bars indicate regions of strong acoustic particle focusing. (**b**) FocuScan with a frequency step of 1 kHz, 1Vpp. Differential impedance peak agrees with the best focusing region (FocuScan minimum).

**Figure 5:**
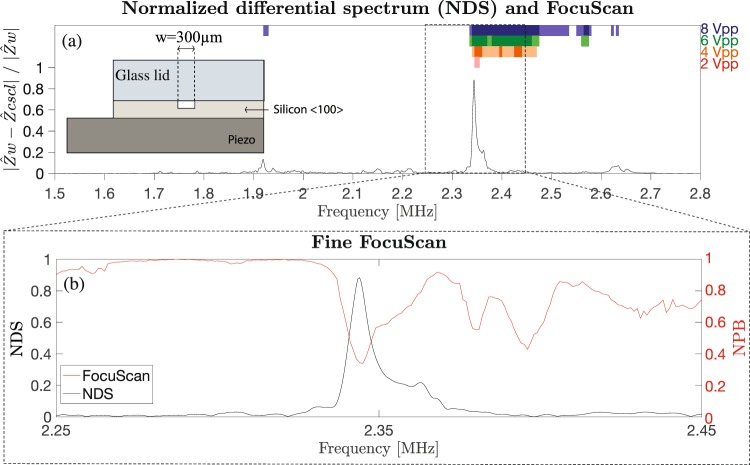
(**a**) Normalized differential spectrum (NDS) water vs 20% CsCl (chip S5). Colored bars at the top indicate the level of focusability measured with the FocuScan method at actuation voltages from 2–8 Vpp. Light color bars indicate moderate acoustic particle focusing, and intense colored bars indicate regions of strong acoustic particle focusing. (**b**) FocuScan with a frequency step of 1 kHz, 2 Vpp. Differential impedance peak agrees with the best focusing region (FocuScan minimum).

Particle focusing experiments were subsequently carried out with the FocuScan method and the results were compared to the obtained peaks in the NDS of water vs 20% CsCl. First, a wide FocuScan from 1.5 MHz to 2.8 MHz with a frequency step of 5 kHz was performed to get a broadband overview of the region of interest. For different applied transducer voltages (2, 4, 6 and 8 Vpp), the normalized particle bandwidth (NPB) was computed. The minima of the NPB indicated the best working frequencies. Two thresholds (0.4 and 0.2) were set in order to distinguish among three different cases: no focusing (NPB > 0.4), moderate focusing (0.4 < NPB < 0.2) and strong focusing (NPB < 0.2) of the particles. The thresholds were chosen based on standard acoustophoretic separation condition where the flow is split in three different branches, often with equal flow in each branch. The ratio of fluid going into the center branch would thus be 0.33, resulting in all particles going into the center branch during strong focusing and still a large fraction of them during moderate focusing. Figures 4a and 5a show the comparison between the NDS and the results of the wide frequency focusing scan experiments for the studied devices G1 and S5 respectively. The figures show the NDS (black solid line) and colored bands, which represent the results of the focusing experiments. The different colors are used to indicate the different actuation voltages (2, 4, 6 and 8 Vpp) while the intensity of the color indicates the level of focusing. Light color bars indicate modest acoustic particle focusing. Intense colored bars indicate regions of strong acoustic particle focusing. It can be noticed that at the lowest actuation voltage, 2 Vpp, particle focusing (intense red colored bands) is found in close proximity to the NDS peak. As expected by increasing the applied voltage, the frequency region of strong particle focusing widens around the NDS peak.

To better resolve the focusing performance vs. frequency, a finer frequency scan with a frequency step of 1 kHz was implemented at a low voltage setting. A frequency range of 200 kHz was chosen in a region of interest, around the NDS peaks. The results for devices G1 and S5 are shown in Figs. 4b and 5b respectively. It can be seen that there is a good match between the NDS peak and the minimum of the normalized particle bandwidth.

**Figure 6:**
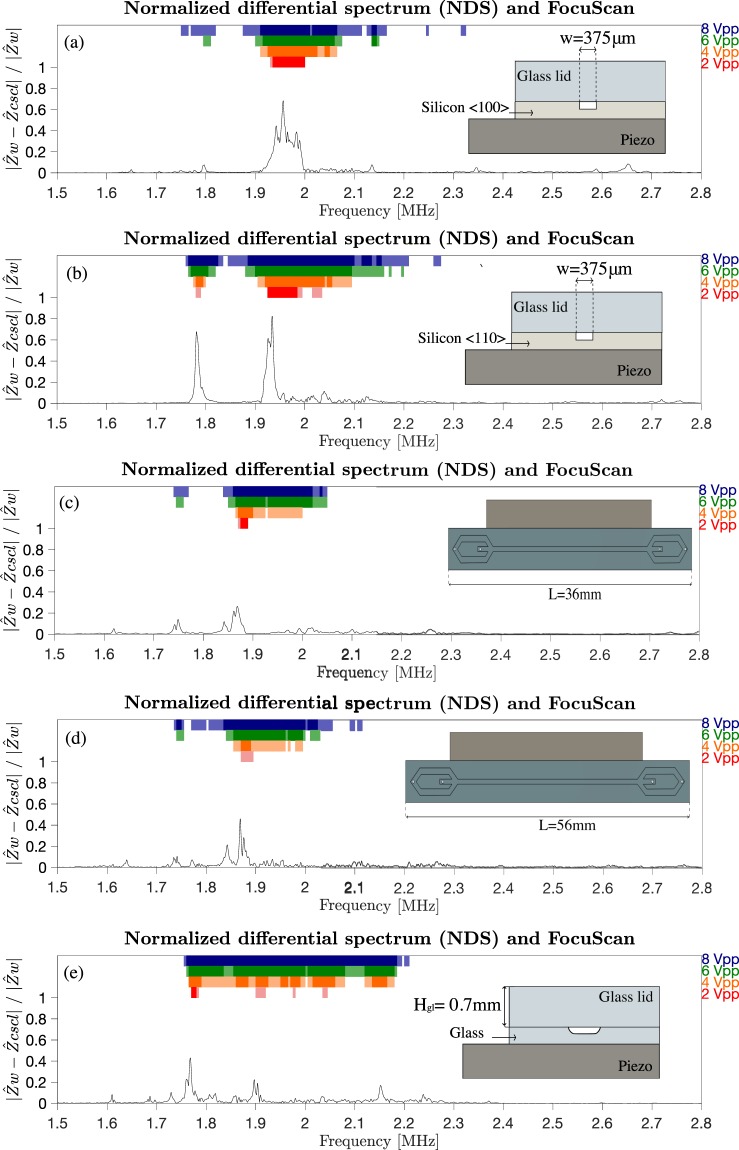
Normalized differential spectrum (NDS) water vs. 20% CsCl for chip (**a**) S1, (**b**) S2, (**c**) S3, (**d**) S4 and (**e**) G2. Colored bars at the top indicate the level of focusability measured with the FocuScan method at actuation voltages from 2–8 Vpp. Light color bars indicate moderate acoustic particle focusing, and intense colored bars indicate regions of strong acoustic particle focusing. Differential impedance peak agrees with the best focusing region. Chip cross-sections or design are also reported. Chip S1(a), S2(b) and G2(e) have a straight channel design. Chip S3(c) and S4(d) have same cross-section as chip S1(a).

A comparison between a wide scan (5 kHz step) and a fine scan (1 kHz step) showed that the resolution did not improve significantly, Supplementary Information Fig. S3, only resulting in a longer scanning procedure. Consequently, the remaining devices were only run in wide frequency scan mode and are shown in Fig. 6a–e.

It can be noticed that at 2Vpp, strong focusing (intense red color) is always found in proximity of the highest peak in the NDS. Furthermore, it can be seen that in other regions of the spectrum where peaks of the NDS are located, particles focus better if the voltage amplitude is increased, e.g. at 1.78 MHz in Fig. 6b. These bulk resonances couple with the λ/2 channel resonance why particle focusing is observed. The matching between the peaks in the NDS and the focusing results reveals that there is a relation between the impedance measurements and the microchannel resonance frequency. In particular, strong focusing is found at the lowest applied voltage at the main peak in the NDS. Thus, the main peak indicates the fundamental resonance frequency of the microchannel. Moreover, the secondary peaks in the NDS permit to identify other resonance modes of the microfluidic channel, at which particles can be focused if the energy introduced into the system is sufficiently increased.

## Conclusions

This paper has demonstrated the feasibility of detecting the optimal working frequency in bulk transversal resonator devices by electrical impedance measurements. The challenge in this study relies on the fact that the acoustic energy in the microchannel represents a minute fraction of the energy in the entire device and the channel impact on the impedance spectra readout is obscured by the resonance modes of the entire system.

Unlike previous studies^30^, we have found the comparison of water vs. air to be non-conclusive. Whereas, the NDS (Normalized Differential Spectrum) for water vs 20% CsCl allows for a precise identification of the microchannel resonance modes as revealed in the system electrical impedance response. The steps defined in the NDS method are:

- Collection of the impedance and phase spectra with water filled and 20% CsCl filled channel;
- Computation of the Normalized Differential Spectrum (NDS);
- The main peak of the NDS indicates the microchannel resonance frequency (Eq. 1.), while smaller peaks indicate other resonance modes;

The method was tested on chips of different design and materials to test the versatility of the NDS method and it turned out to be successful in defining the best working frequencies for all chip configurations studied. The differential impedance spectra method enables a simple way to determine the optimal working conditions for bulk transversal resonator devices.

## Materials and Methods

### Acoustophoretic devices

To evaluate the feasibility of detecting the optimal working frequency through differential impedance spectra measurements, seven devices of different designs and materials were tested. The devices were grouped in two different types marked by a capital letter: (S) silicon-glass devices and (G) glass-glass devices. To distinguish devices of the same category, a different number was placed next to the group letter.

Devices S1–S4 were fabricated in the cleanroom facilities at Lund University using conventional silicon microfabrication techniques^28^. Flow channels were wet etched in ~380 μm thick, 3-in., <100> and <110> silicon wafers rendering a rectangular cross section resonator channel (375 μm wide and 150 μm deep). Fluid access holes were etched from the back side of the chip. The wafers were diced, and 1.1 mm-thick borosilicate glass was anodically bonded to the silicon to seal the channel structure.

Chip S5, G1 and G2 were purchased from an external manufacturer (Micronit Microtechnologies B.V., Netherlands). Chip S5 was fabricated using deep reactive ion etching, giving a 300 μm wide and 150 μm deep flow channel. Devices of type G were fabricated by standard UV lithography and wet etching of borosilicate glass (thickness: 0.4 mm). The chips were bonded to a borosilicate glass lid (1.1 mm-thick for G1, 0.7 mm thick for G2) to form a sealed device. When wet etching glass, the channel cross section has a semicircular shape^39^.

The cross sections and the different designs of devices of type S and G are shown in Supplementary Fig. S4. In Supplementary Table S1 the main differences in chip dimensions, piezo dimensions and design are highlighted.

The chips were actuated using a 2 MHz thickness mode piezoelectric transducer (PZ26, Ferroperm Piezoceramics, Denmark) glued (Loctite^R^ Bruch-on Super Glue, Henkel Corporation, Connecticut) to the backside of the devices. Silicone tubing was attached to each inlet and outlet using silicone glue (ELASTOSIL A07, Wacker, Germany).

### Impedance measurements

Impedance and phase measurements were carried out using an impedance analyzer (HP 4194 A Impedance/Gain – Phase Analyzer, Hewlett-Packard, USA). The instrument was connected to a computer and controlled using MATLAB 2014. A frequency sweep from 1.5 MHz to 2.8 MHz was carried out with a frequency step of 0.5 kHz. The instrument was set to give the average of eight consecutive measurements.

Two different analyses were performed using this setup. Firstly, it was investigated how the impedance spectrum changed while assembling the devices. The assembling steps that were evaluated are: (1) the free piezoelectric transducer and (2) piezoelectric transducer attached to a water-filled chip. Secondly, it was studied if it was possible to get information about the microchannel from the electrical response of the system. Each device was thus connected to the impedance analyzer through the wires soldered on the piezoelectric transducer. Impedance measurements were carried out under three conditions: (1) air filled channel, (2) distilled water filled channel and (3) 20% Cesium Chloride (CsCl, a density modifying salt) filled channel. Impedance measurements were consecutively recorded for the different conditions.

The density and speed of sound of water and the solution of 20% CsCl were measured using a density meter (DSA 5000 M, Anton Paar, Graz, Austria), as shown in Supplementary Table S2. The corresponding properties of air are also reported.

### Focusing experiments

Teflon tubing was inserted in the silicone tubing and each device was placed under a microscope (Leica DM2500 M, Leica Microsystems IR GmbH, Germany). A camera (Infinity1–2, Lumenera, Canada) was used to clearly visualize the microchannel, specifically the end part of the separation channel. A solution of water and 4.8 μm polystyrene particles (Fluoro-Max Dyed Green Aqueous Fluorescent Particles, Thermo Scientific, USA) was perfused through the microchannel using a 1 ml syringe and a syringe pump (SP210IWZ Syringe Pump, World Precision Instruments, Inc., Germany). The pump rate was set to 40 *μl*/*min*. This resulted in Reynolds number of 5, well within the laminar regime.

An in-house developed automatic setup, FocuScan, was used to carry out the particle focusing experiments (see Supplementary Information Fig. S5). This setup permitted to identify the best working frequency by an automatic actuation of the device, acquisition of microbead focusing images and image post-processing. More specifically, a LabVIEW program (National Instruments Corporation, USA) was used to operate the device through Analog Discovery 2 (Digilent, USA) and a home-made amplifier. Using the FocuScan software, it was possible to perform a frequency sweep, setting up the start and end frequency, the frequency step and the delay time between steps. Two frequency sweeps were carried out for each tested device. Firstly, a wide frequency sweep (wide FocuScan) from 1.5 MHz to 2.8 MHz with a frequency step of 5 kHz and a delay time between sweeps of 4 s. The wide FocuScan was repeated for different peak-to-peak voltages (2, 4, 6 and 8 Vpp). Secondly, a finer frequency scan (fine FocuScan) was carried out in a region of the spectrum of interest, typically a 200 kHz range, with a frequency step of 1 kHz and a delay time between sweeps of 4 s. The fine FocuScan was performed with a low set voltage amplitude (1 or 2 Vpp depending on the device).

**Figure 7:**
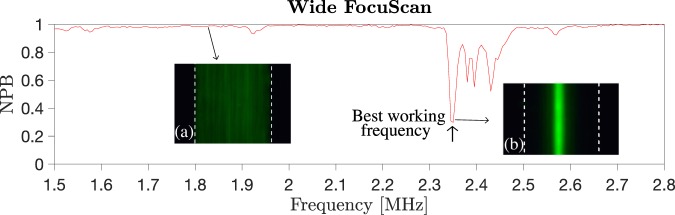
Example of the wide FocuScan output (chip S5). The plot shows the normalized particle bandwidth (NPB) vs actuation frequency. The NPB is close to 1 when particles occupy the whole width of the microchannel. (**a**) When particles are acoustically focused, the NPB gets smaller. (**b**) The minimum of the FocuScan indicates the best working frequency. White dashed lines indicate the channel boundary.

Additionally, LabVIEW was used to control the image acquisition. A total of ten images were acquired and saved at each frequency step. The images were subsequently post-processed using MATLAB 2014. First, an average of the images acquired at each frequency step was performed. For each average image, and thus for each actuation frequency, the full width at half maximum of the focused particle band was then computed and normalized over the width of the microchannel. In this way, the normalized particle bandwidth (NPB) was calculated. The NPB is a number between 0 and 1. When the particles occupy the full width of the microchannel, the NPB is close to 1. Whereas, when the particles experience the radiation force, they are moved to the center of the microchannel and the NPB is reduced. In order to identify the best working frequency, the NPB vs actuation frequency was plotted. The minima of the NPB indicated the best working frequencies (Fig. 7).

## Supplementary information

Supplementary Information

## Data Availability

The datasets generated and analyzed during the study are available from the corresponding author on request.
