# Supplementary material for: Differential impedance spectra analysis reveals optimal actuation frequency in bulk mode acoustophoresis
Source: Sci Rep. 2019 Dec 13;9:19081. doi: 10.1038/s41598-019-55333-1 (PMC6911075; doi:10.1038/s41598-019-55333-1)
Supplement: Supplementary file 1 — Supplementary Information [file 41598_2019_55333_MOESM1_ESM.pdf]

Supplementary material

Differential impedance spectra analysis reveals optimal actuation frequency in bulk mode acoustophoresis

Valentina Vitali, Giulia Core, Fabio Garofalo, Thomas Laurell, Andreas Lenshof

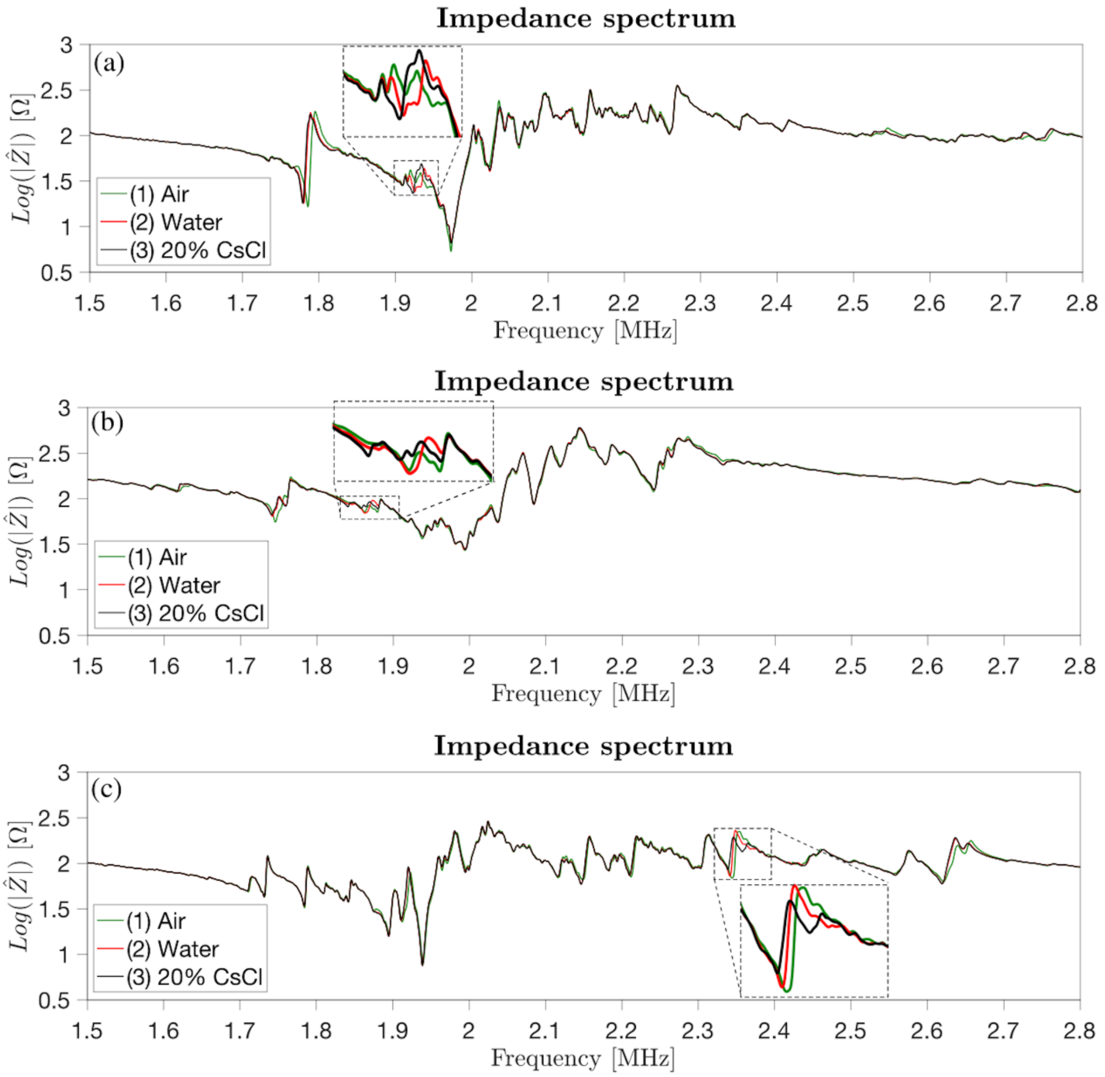

Figure S1: Impedance magnitude spectrum of the device (a) S2, (b) S3 and (c) S5 with different media inside the microchannel: (1) air, (2) water and (3) 20% CsCl solution. The zoom – in inserts highligths the region of the spectra where there is a clear spectral deviation for the different channel media. The deviation can be seen as a frequency shift and is related to the changed resonance frequency linked to the corresponding media loaded in the microchannel.

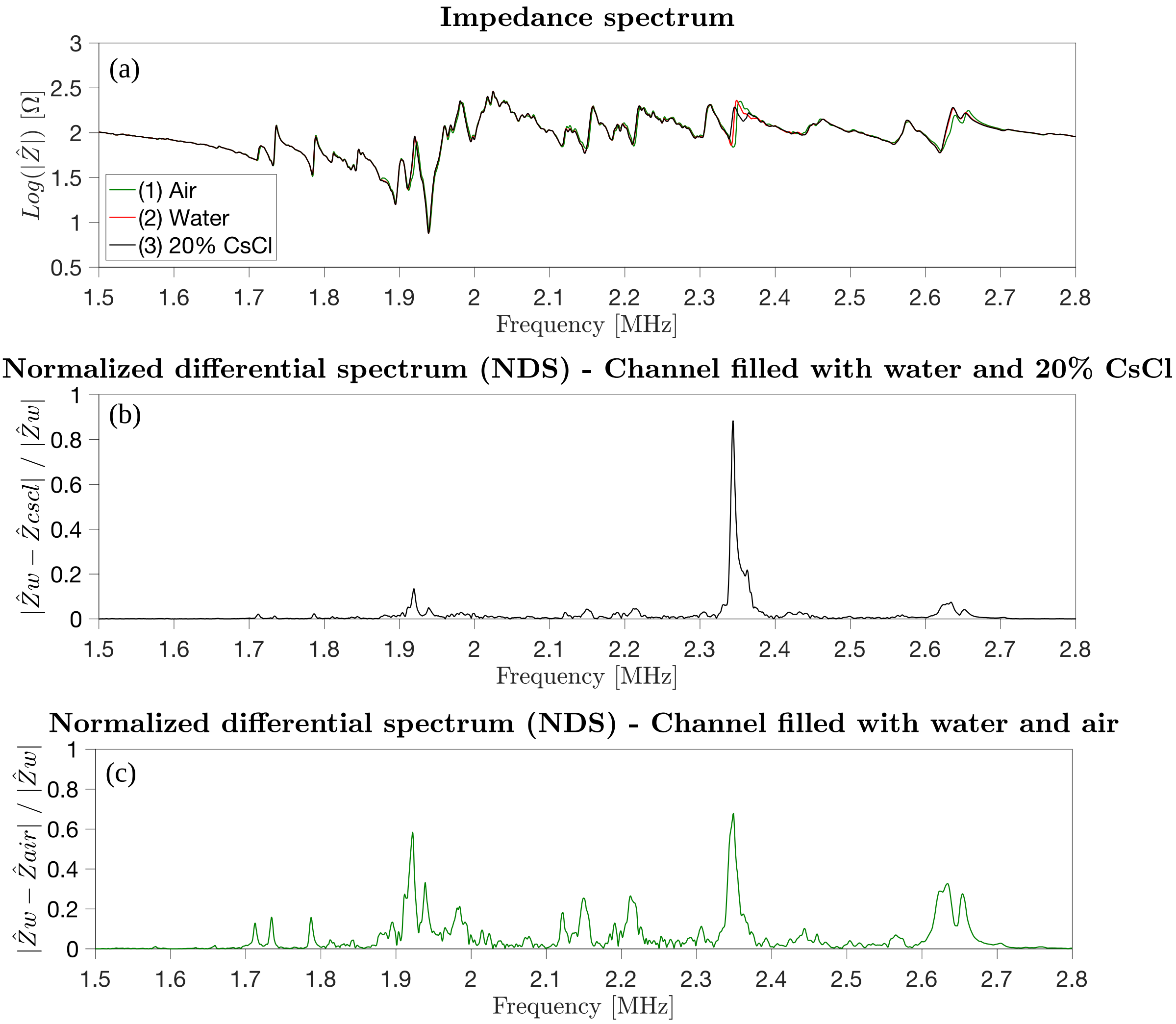

Figure S2: (a) Impedance magnitude spectrum of a (1) air filled, (2) water filled and (3) 20% CsCl filled device (chip S5). (b) Normalized Differential Spectrum (NDS) water vs 20% CsCl. (c) NDS water vs air. It can be seen that the NDS water vs 20% CsCl presents a clear peak at 2.35 MHz. The same information can be derived from the NDS water vs air although more spurious peaks are seen.

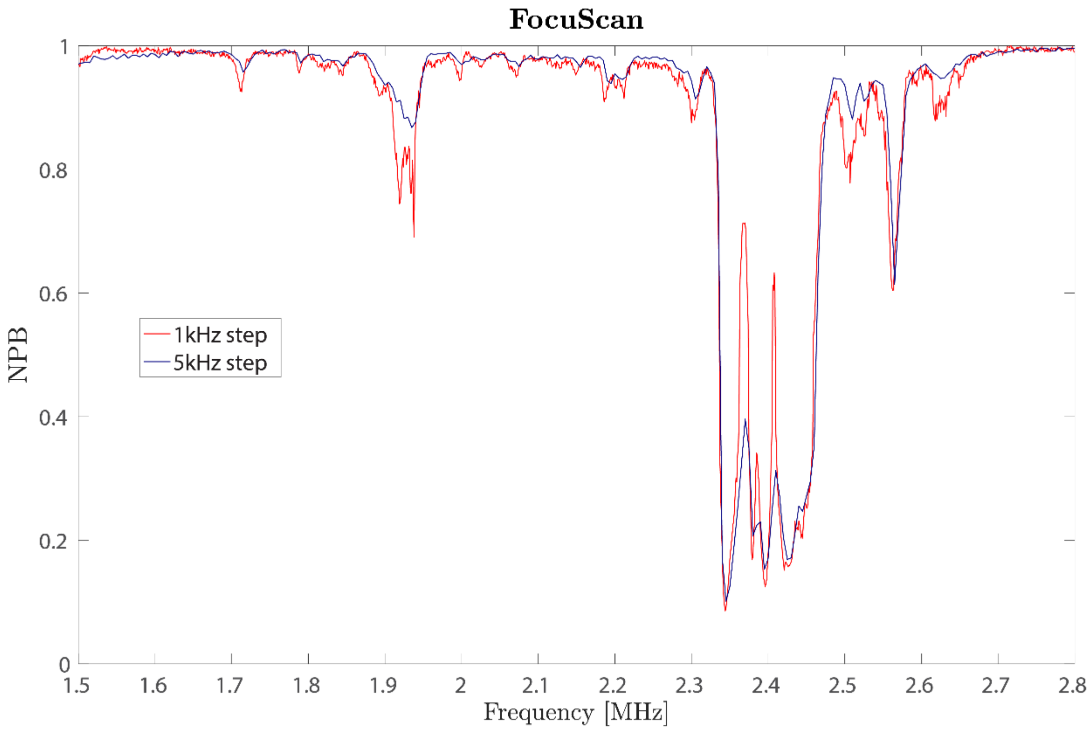

Figure S3. Comparison of the wide scan mode (5kHz step) and the fine scan mode (1kHz step). It can be seen that the fine scan indeed provides more information, the large peaks are clearly visible in both cases, but the useful information is the same and is obtained at a much faster pace with the wide scan compared to the fine scan.

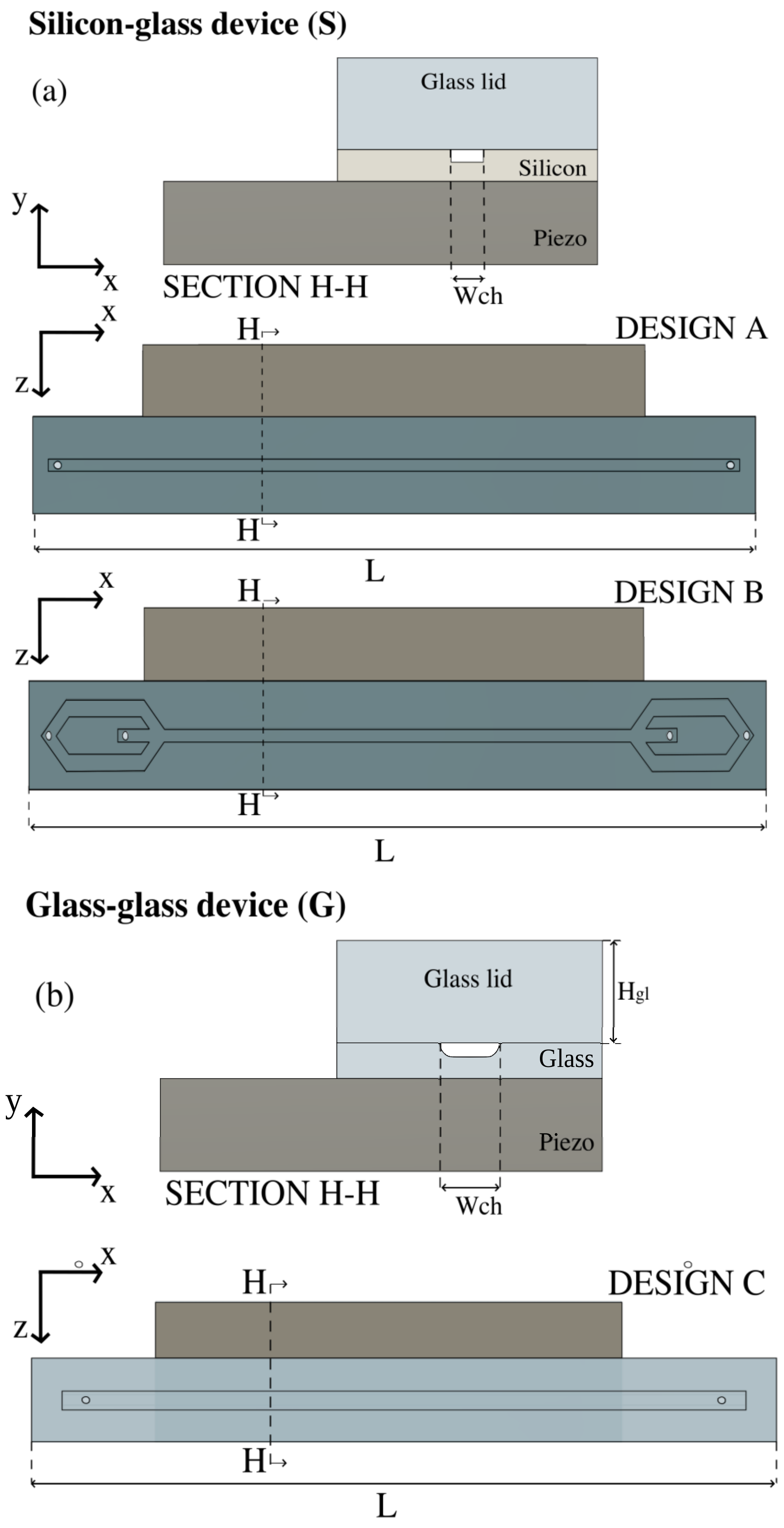

Figure S4: Sketch of the cross-section and of the designs of (a) silicon-glass devices (type S) and (b) glass-glass devices (type G). The microchannel width (Wch), the glass lid height (Hgl) and the substrate length (L) are indicated.

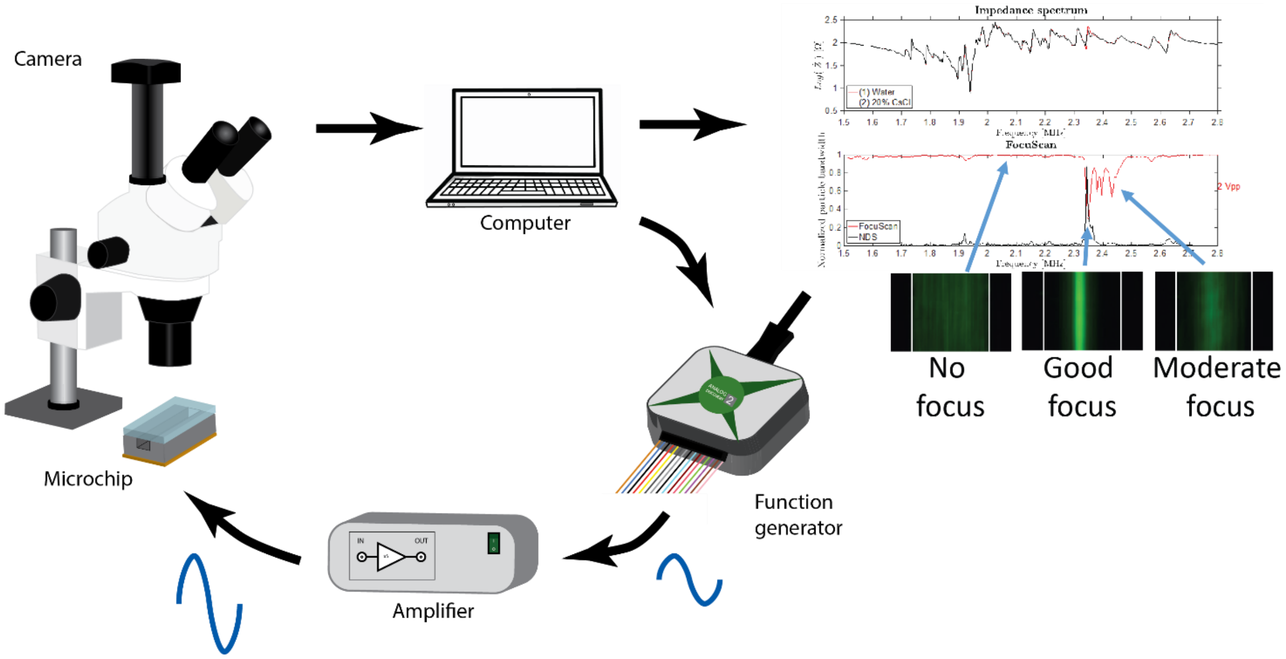

Figure S5: Schematic of the FocuScan setup.

Table S1: Main differences in dimensions and design among the tested devices. To distinguish devices of the same category a number is placed next to the category letter. The channel width (Wch), silicon orientation, glass lid height (Hgl), substrate length (L) and piezo dimensions are reported. For devices of type G, Wch indicates the widest part of the channel.

**Silicon-glass devices (type S)**					
	**Wch [µm]**	**Silicon orientation**	**L [mm]**	**Piezo dimensions [mm]**	**Design**
S1	375	<100>	40	25x5x1	A
S2	375	<110>	40	25x5x1	A
S3	375	<100>	36	15x5x1	B
S4	375	<100>	56	25x5x1	B
S5*	300	<100>	40	25x5x1	A
**Glass-glass devices (type G)**					
	**Wch [µm]**	**Hgl [mm]**	**L [mm]**	**Piezo dimensions [mm]**	**Design**
G1*	410	1.1	44	25x5x1	C
G2*	410	0.7	44	25x5x1	C

* *Fabricated by Micronit Microtechnologies B.V., Netherlands*

Table S2: Densities and speed of sound of distilled water, 20% CsCl solution and air given at T = 25°C.

	Density ρ [kg m^{-3}]	Speed of sound c [m s^{-1}]
Water	998,480	1498,96
20% CsCl	1149,686	1486,78
Air	1,184	346
